# Des furoncles résistants aux antibiotiques: penser à la myiase !!

**DOI:** 10.11604/pamj.2013.15.41.2621

**Published:** 2013-06-04

**Authors:** Faida Ajili, Rim Abid, Najeh Bousseta, Ali Mrabet, Ghazi Karoui, Bassem Louzir, Riadh Battikh, Salah Othmani

**Affiliations:** 1 Service de médecine interne. Hôpital militaire de Tunis. Montfleury 1008, Tunisie

**Keywords:** Furoncles, myiase, mouches, Furuncles, myiasis, flies

## Abstract

Les myiases sont des infections parasitaires par des larves de mouches. La localisation cutanée doit être évoquée de retour d'un pays tropical devant une évolution inhabituelle de lésions cutanées. Nous rapportons une observation d'un militaire tunisien, ayant séjourné en République Démocratique du Congo. Il était atteint de myiase cutanée simulatrice d'une furonculose résistante aux antibiotiques. L'intérêt de cette observation est de souligner l'importance d’évoquer la myiase dont le traitement est simple et rapide chez un patient de retour de zone d'endémie.

## Introduction

Les myiases (du grec myia = mouche) sont des zoonoses résultant de l'infestation de l'homme ou d'animaux vertébrés par diverses espèces de larves de diptères. La symptomatologie clinique est variable selon la localisation de l'atteinte: cutanées, sous cutanées, cavitaires, ou des conduits naturels. Nous rapportons une observation d'un militaire tunisien atteint de myiase cutanée simulatrice d'une furonculose.

## Patient et observation

**Figure 1:**
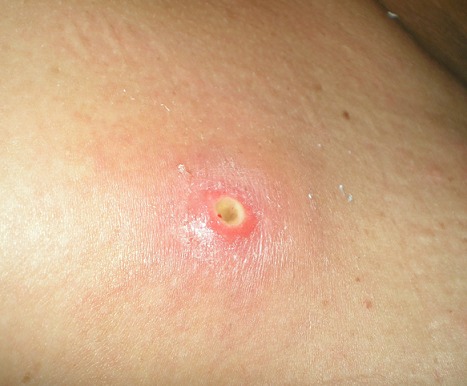
Lésion furonculoïde de l’épaule, entourée d'un liseré érythémateux légèrement tuméfié

Il s'agit d'un militaire tunisien, âgé de 37 ans, de sexe masculin, en détachement onusien au contingent tunisien en République Démocratique du Congo durant l'année 2008. Dans ses antécédents, il y a un diabète non insulinodépendant bien équilibré sous antidiabétiques oraux depuis cinq ans et une obésité (Indice de Masse Corporelle =37 kg/m^2^) associée à une dyslipidémie. En octobre 2008, ce militaire était déployé pendant 10 semaines dans une zone périphérique de Kinshasa. Un mois après, il s′est présenté à la consultation, souffrant de deux papules prurigineuses isolées apparues depuis 6 jours au niveau du thorax et de l’épaule. L'examen clinique a trouvé deux lésions furonculoïdes, de 5 mm de diamètre chacune, avec un petit orifice ne laissant pas sourdre du pus, entouré d'un liseré érythémateux légèrement tuméfié, sans chaleur locale (Figure 1). Le reste de l'examen était sans particularité notamment pas de fièvre et le bilan biologique standard n′apportait pas d′élément d′orientation. Devant le terrain de débilité (diabète type II), un traitement à base d'oxacycline à la dose de 2 g/jour pendant 7 jours per os avec des soins locaux a été instauré mais s'est avéré inefficace. Le retard de guérison fut d'abord rattaché à son diabète et les lésions ont été traitées par une pommade antibiotique à base de cyclines (pommade hydrophobe). Après deux jours de traitement par la pommade grasse, deux larves blanchâtres de dimensions 10x5mm ont sailli de chaque lésion à la suite d'une compression bidigitale. Ces larves ont été retirées facilement à l'aide d'une pince, laissant derrière elles un orifice propre sans pus. L'aspect furonculoïde de la lésion cutanée, la chronologie des événements, la notion de séjour en république Démocratique du Congo, confrontés à la morphologie de la larve ont permis de conclure au diagnostic de myiase furonculoïde et les larves étaient identifiées comme appartenant à *Cordylobia anthropophaga*.

## Discussion

La myiase désigne l'ensemble des troubles provoqués par la présence dans un corps humain ou animal de larves de diptères parasites des familles Calliphoridae, Sarcophagidae, Cuterebridae, Muscidae. Le développement actuel des voyages intercontinentaux à visée professionnelle ou de loisirs avec séjours dans la jungle entraînent un accroissement du nombre de cas de myiases importés dans les pays non endémiques. La forme qui sévit en Afrique est surtout due au Cordylobia anthropophaga (Diptera Calliphoridae) comme dans notre cas [1].

Sur le plan clinique, on distingue: La myiase cutanée, parfois furonculaire (objet de notre observation), la myiase cavitaire (ou luminale) et la myiase intestinale qui reste exceptionnelle. Les Myiases furonculaires sont des infestations cutanées et sous-cutanée endoparasitaires, causées par une ou plusieurs larves de diptères dont les larves ectoparasites sont pondues directement par la mouche sur la peau ou sur un vecteur secondaire (tel que: moustique, tique’) ou au sol et sur les tissus [2, 3]. Ces larves ne s'attaquent qu'aux zones cutanées mais peuvent s'enfoncer jusqu’à 1 cm voire un peu plus sous la peau. La présence de la larve se manifeste généralement par un furoncle ou un kyste sébacé, souvent surinfecté et ne réagissant pas au traitement antibiotique. Quand elle grandit, la larve est parfois perçue par son hôte (sensation de mouvement sous la peau), mais pas toujours constante. La réaction immunitaire de l'organisme ne chasse pas la larve, mais marque son emplacement d'une papule rougeâtre, puis d'un nodule cutané percé en son centre pour permettre à la larve de respirer. Grâce à cette ouverture, entretenue par la larve, on peut quand la larve est assez grosse, l'enlever en la tirant délicatement avec une pince à épiler ou au moyen d'une seringue spéciale aspirant le venin [4]. En raison de son aspect clinique furonculoïde, la lésion est souvent confondue avec d'autres dermatoses d'origine infectieuse comme un abcès bactérien, une infection à mycobactérie (tuberculeuse ou non), une infection fongique, une actinomycose. La lésion peut également être confondue avec un kyste épidermoïde ou sébacé surinfecté ou avec une tungose [5]. En pratique le diagnostic clinique est suffisant dans la plupart des cas pour un ‘il exercé. Si une histologie est faite, elle montrerait une forme larvaire cylindrique, facilitant son extraction manuelle [6].

Dans le cas décrit ici, le tableau est celui de myiase furonculeuse, avec un aspect de tuméfaction rouge et douloureuse pseudofuronculeuse. L'association d'un tel aspect à la résistance aux traitements, la chronologie des événements, le contexte épidémiologique avec la notion de séjour en république Démocratique du Congo, confrontés à la morphologie de la larve ont permis de conclure au diagnostic de myiase furonculoïde et les larves étaient identifiées comme appartenant à *Cordylobia anthropophaga*.

La Prise en charge curative consiste à asphyxier la larve durant la phase initiale de l'infection en bouchant l'ouverture de son gîte par un corps gras durant environ 48 heures. La larve cherche à remonter en surface pour respirer. Elle traverse la couche de lard où on la piège alors (le lard est parfois remplacé par une épaisseur de coton imprégnée de vaseline) [7]. Certains préfèrent attendre quelques jours pour que la mouche émerge spontanément sous sa forme adulte. Un traitement antibiotique par voie orale et un vaccin antitétanique de rappel sont souvent recommandés pour limiter le risque de surinfection bactérienne. Chez notre patient, les suites ont été simples après l'extraction de ces larves avec une cicatrisation complète en quelques jours.

Sur le plan préventif, certains moyens d′application simple, peuvent prévenir ces parasitoses [2]: le trempage des draps et des habits dans de l'eau bouillante avant de les utiliser, le repassage au fer très chaud des tissus qui seront en contact avec le corps ou le bouchage des orifices du lit de camp avec de la cire ou du savon.

## Conclusion

La myiase cutanée, actuellement disparue du quotidien de nos consultations, reste une infection du voyageur dans les zones tropicales. Devant des lésions furonculoïdes il faut penser à cette parasitose chez ces patients. Le traitement de cette infection est simple mais astucieux. Son évolution est bénigne.
